# IgG Antibody Titers Against *Ascaris lumbricoides*, *Strongyloides stercolaris*, and *Toxocara canis* in Venezuelan Patients with Asthma or COPD

**DOI:** 10.3390/tropicalmed9110253

**Published:** 2024-10-24

**Authors:** Juan Bautista De Sanctis, Dolores Moreno, Nancy Larocca, Jenny Valentina Garmendia

**Affiliations:** 1 Institute of Immunology Dr. Nicolás E. Bianco, Faculty of Medicine, Universidad Central de Venezuela, Caracas 1050, Venezuela; laroccanancy@gmail.com (N.L.); jenny.garmendia@gmail.com (J.V.G.); 2 Institute of Molecular and Translational Medicine, Faculty of Medicine and Dentistry, Palacky University, Hněvotínská 1333/5, 77900 Olomouc, Czech Republic; 3 Chair of Physiopathology, Luis Razetti School of Medicine, Faculty of Medicine, Universidad Central de Venezuela, Caracas 1050, Venezuela; moreno.d1@gmail.com

**Keywords:** COPD, asthma, atopy, eosinophils, IgE, *Ascaris lumbricoides*, *Giardia lamblia*, *Strongyloides stercolaris*, *Toxocara canis*

## Abstract

It has been suggested that parasitic infections, common in Latin American populations, may amplify the inflammatory response of the airways. There are several reports of atopic and asthmatic patients but few reports of parasitic infection in COPD patients. This study aimed to determine the prevalence of parasitic infections in COPD patients compared with atopic and asthmatic patients attending the Institute of Immunology outpatient clinics and the pneumology service of the University hospital. A case-control study was conducted compising 100 patients with bronchial asthma, 100 patients with COPD, 100 individuals with atopy without respiratory symptoms, and 100 healthy individuals. Serum-specific IgG antibodies against the parasites *Ascaris lumbricoides* (*Al*), *Strongyloides stercolaris* (*Ss*), and *Toxocara canis* (*Tc*) were measured by ELISA. IgE levels were used as an indirect indicator of atopy. Positive IgG for *Al* was observed in all groups, predominantly in the atopic cohort; *Ss* positiveness was recorded only in four COPD patients, and *Tc* positiveness was observed in all groups except in controls. Significant correlations exist between the values of *Al* and IgE in controls, atopic, and asthmatic patients without COPD. No correlation was found for *Tc*. IgE levels and the forced expiratory volume in 1 s (FEV1) correlate only in atopic and asthmatic patients. Parasitic infections are common in atopic patients and moderate and severe asthmatic and COPD patients. Anti-inflammatory treatment may be responsible for the increased frequency of infection in moderate and severe asthmatic and COPD patients.

## 1. Introduction

Chronic obstructive pulmonary disease (COPD) is a long-term inflammatory lung condition in which airflow is partially reversible [1,2,3,4]. Its symptoms include difficulty breathing, coughing, mucus production, and wheezing [1,2,3,4]. COPD is mainly caused by prolonged exposure to irritating gases or particulate matter, usually from cigarette smoke [1,2,3,4]. COPD patients are at higher risk of developing heart disease, lung cancer, and other conditions [1,2,3,4]. COPD can also be related to asthma, i.e., COPD asthma overlap syndrome. Patients with COPD asthma overlap syndrome may differ from classical COPD patients in clinical manifestations and response to treatment.

Emphysema and chronic bronchitis are the two most common conditions contributing to COPD [1,3,4,5]. These two conditions usually occur together and can vary in severity among individuals with COPD [1,3,4,5]. Chronic bronchitis is the inflammation of the lining of the bronchial tubes, which carry air to and from the air sacs (alveoli) of the lungs. It is characterized by a daily cough and mucus (sputum) production. Emphysema is a condition in which the alveoli at the end of the lungs’ smallest air passages (bronchioles) are destroyed due to damaging exposure to cigarette smoke and other irritating gases and particulate matter. Although COPD is a progressive disease that worsens over time, it is treatable. With proper management, most people with COPD can achieve reasonable symptom control and quality of life, as well as reduce the risk of other associated conditions.

In recent years, it has become evident that a subgroup of COPD patients are atopic patients. In these patients, there is a need to control allergic reactions, which may increase respiratory inflammation. Type 2 inflammation assessment is also a part of the standard clinical control of COPD patients [6]. In general, the level of eosinophils in the blood is higher in COPD patients than in healthy subjects, although there is marked variability. Higher blood eosinophil values in patients with COPD are associated with a higher number of eosinophils in the airways [3,6]. Eosinophil count may explain the response to glucocorticoid treatment [3,7]. Moreover, peripheral blood eosinophil levels seem to define a type of COPD patient that appears to respond better in exacerbations, although it is more prone to be hospitalized [8,9]. In current guidelines for treating COPD, the blood eosinophil count is part of the initial assessment after diagnosing and treating COPD [3].

Asthma is a chronic lung condition characterized by recurring symptoms, reversible airflow obstruction, and easily triggered bronchospasms [10,11,12]. It is also a chronic respiratory condition characterized by symptoms like dyspnea, cough, and wheezing [10,11,12]. As spirometry shows, diagnosis requires consistent symptoms and variable expiratory airflow obstruction [10,11,12]. Treatment aims to control symptoms and prevent exacerbations based on symptom frequency and severity [10].

The development of asthma, often starting in childhood, involves a complex interaction of genetic and environmental factors related to atopy [10,11,12]. Researchers are working on predictive systems to identify individuals at risk of symptoms into adulthood [13,14,15]. Despite advancements in genetic and environmental factors, clinical strategies are lacking to reduce persistent asthma development [13,14,15,16]. This covers epidemiology, pathophysiology, assessment, pharmacological treatment, and monitoring strategies for adolescents and adults, aligning with recommendations from asthma organizations [10].

Intestinal nematode infections, particularly the most common one caused by the roundworm *Ascaris lumbricoides*, affect up to one-third of the global population. [17,18]. Humans can become infected with *A. lumbricoides* by ingesting their eggs, often found in food contaminated by human feces [17,18]. Infection can also occur by consuming contaminated dirt from hands or fingers. Once ingested, *A. lumbricoides* eggs hatch in the duodenum, and the resulting larvae penetrate the small bowel wall, migrating through the portal circulation to the heart and lungs [17,18]. The larvae then lodge in the alveolar capillaries, penetrate alveolar walls, and ascend the bronchial tree into the oropharynx. Following this, they are swallowed and return to the small bowel, where they develop into adult worms, mate, and release eggs into the stool [17,18]. The life cycle is completed in approximately 2 to 3 months, while adult worms have a lifespan of 1 to 2 years [17,18,19]. Migration of Ascaris larvae through the lungs may cause symptoms such as cough, wheezing, and occasionally hemoptysis or other respiratory symptoms in individuals without exposure to Ascaris.

The diagnosis of ascariasis is typically confirmed by the microscopic detection of eggs in stool or the observation of adult worms in stool or emerging from the nose or mouth. Occasionally, larvae can be found in sputum during the pulmonary phase. Eosinophilia can be prominent during larvae’s migration through the lungs [20,21], and it can diminish when adult worms reside in the intestine. While most cases of ascariasis are asymptomatic, infection and reinfection can occur in areas with poor sanitation and are associated with malnutrition, iron deficiency anemia, and stunted growth and cognitive development [17,18,19]. Aside from eosinophil count, IgE antibodies against *Ascaris lumbricoides* suggest that the infection is related to allergic asthma, since parasite antigens can induce bronchoconstriction in exposed individuals [22]. Treatment is primarily symptomatic in cases where the lungs are affected and may include bronchodilators, corticosteroids, and anthelmintic drugs.

Strongyloidiasis is an intestinal infection caused by the parasitic nematode *Strongyloides*, primarily affecting humans through *S. stercoralis* [23]. This species can persist within a host for extended periods without causing symptoms in healthy individuals. However, instances of hyperinfection syndrome have been documented [23,24,25,26,27]. The larvae enter the host through the skin and migrate directly to the lungs via blood vessels, inducing inflammation and exacerbating existing disease. As they reach the alveolar space, the larvae progress through the trachea and pharynx before being swallowed [23,24,25,26,27]. In cases of hyperinfection, a large number of larvae can invade the bloodstream, lungs, central nervous system, and other organs, resulting in the disruption of the intestinal mucosa and the presence of bacteria on the surface of invading larvae [25].

Strongyloidiasis can cause life-threatening infections in immunocompromised individuals [23,24,25,26,27]. Symptoms include watery diarrhea, abdominal cramping, and urticarial rash [23,24,25,26,27]. During chronic uncomplicated infections, the larvae may migrate to the skin, causing cutaneous strongyloidiasis, known as larva currens, due to the larva’s quick migratory rate [23,24,25,26,27]. The parasite can reinfect individuals if not adequately treated [23,24,25,26,27].

Toxocariasis is a widespread zoonotic disease that humans can contract from dogs, cats, and wild hosts [28,29,30]. Infection with *T. canis* in humans can result in toxocariasis. The primary mode of human infection, particularly in individuals under 20 years of age, is ingesting eggs from feces-contaminated objects. While uncommon, other potential sources of infection include close contact with infected animals, exposure to soil containing infectious eggs, handling soil with open wounds, accidental ingestion of contaminated soil, and consumption of undercooked or raw meat from intermediate hosts such as lamb or rabbit [29,30]. Neurological and ocular manifestations are frequently observed in Toxocara infections. Additionally, allergic manifestations have been documented [31], along with pulmonary involvement [32,33].

Diagnosing most parasitic infections relies on stool analysis of the parasite or eggs [34,35,36]. However, in humans, *Toxocara canis* infection has to be analyzed by serological methods [37,38]. Parasite detection in stool may require the analysis of serial samples to define infection since the sensitivity of the standard Kato–Katz method is low. In recent years, detecting parasite antigens in the stool has facilitated diagnosis. Moreover, detecting antibodies in serum against specific antigens has amplified and simplified the analysis of parasitemia and follow-up of patients. Considering the complexity of analysis in a large population of individuals attending the outpatient clinic of the Institute of Immunology and the pneumology service at the Central University Hospital in Caracas, we decided to analyze the levels of IgG antibodies against the three most relevant parasites that can be critical to screen in patients with asthma, COPD, and atopic individuals. The objective is to analyze the presence of IgG using commercial standardized kits to analyze a possible correlation with forced expiratory volume in 1 s (FEV1), hematological and IgE values, and disease severity (asthma and COPD).

## 2. Materials and Methods

### 2.1. Human Samples

The case-control study comprised patients who had attended the outpatient clinic for at least one year. The patients exhibited no anomalies in paraclinical analysis, signifying the absence of infectious or exacerbating diseases. The cohort’s admixed ancestry was previously defined [39]. Pregnant women, patients with cancer or other comorbidities, cardiovascular diseases, diabetes, and autoimmune disorders were excluded. The cohort had four groups:

1. The control group: A total of 100 individuals without atopy, normal spirometry, typical hematological values, and normal IgE levels were included.
2. The atopic group included 100 patients with clinically defined atopy without lung involvement [40]. The group consisted of individuals with skin and food allergies; only 20 had mild chronic rhinitis without obstructive components.
3. The asthma cohort: A total of 100 patients were classified according to GINA guidelines [10,11]. The patients with mild and intermittent patients had a predicted FEV1 > 80% and with few episodes per month. The moderate patients were on continuous treatment with a short beta-agonist and a predicted FEV1 > 60%, and the severe patients with predicted FEV1 < 60% were treated with local steroids plus a short-term beta agonist. All patients used inhaled steroids and eventually salbutamol for rescue.
4. The COPD cohort of 100 patients was classified according to the GOLD guidelines [1,2,3]. Patients were stable at the time of sample collection, and there were no recent exacerbations. The mild patients had an expected FEV1 > 80%, the moderate group had a predicted FEV1 > 50 but <80, and the severe group had a predicted FEV1 between 40 and 50. Depending on the severity, the treatment was inhaled corticosteroid, long-acting ẞ2-agonist, and/or long-acting muscarinic antagonist.

Two 5 mL samples were taken in heparin for hematologic analysis and a dry tube for serum. The blood samples were taken when patients attended control medical checkups. Hematological counts were performed using the Beckman Coulter Ac.T Diff hematology analyzer. The serum of each sample was obtained after 30 min of sample collection by centrifuging clotted venous blood. The serum was stored at −70 degrees until the ELISA analysis was performed. The three ELISA kits were used to avoid sample freezing and thawing, which may alter the titters of IgG.

Written consent was obtained from all individuals interested in participating in the study. This study received approval from the Ethical Committee of the Institute of Immunology, Faculty of Medicine, Caracas, Venezuela (approval number 20052308).

### 2.2. ELISA Kits and Result Analysis

Diagnosis of parasitic infection mainly relied on commercial immunological techniques. For *Ascaris,* the common ABA-1 antigen is used for screening; for *Strongyloides*, the antigen from *S. papillosus* larvae antigen is used; and for *T. canis* larval excretory–secretory antigen-based ELISA (TcES-ELISA) is used. The following commercial kits were used: (1) MyBioSource for *Ascaris lumbricoides* (*Al*), (2) Abcan for *Toxocara canis* (*Tc*), and (3) Euroimmun for *S. stercolaris (Ss)*.

The tests were performed according to the manufacturer’s guidelines. For the Mybiosource kit, the sera were diluted (1:100 *v*/*v*) with the buffer supplied by the provided, and it was added to the already treated ELISA plates. Five assay controls were used: a negative control, a certified pooled sample from certified negative individuals, a negative control of the kit, a positive control of the kit, and a certified sample from the Institute of Tropical Medicine, Faculty of Medicine. Universidad Central de Venezuela. In addition, the stool samples of the positive patients were analyzed by expert technicians of the Institute of Tropical Medicine using standard techniques (see Section 2.3).

For the Abcan kit to assess *T. canis*, the sample dilution was carried out at 1:100, as suggested by the manufacturer, and the certified positive and negative controls were used along with the controls from the kit. The positive individuals were certified by the analysis performed by the Institute of Tropical Medicine, Faculty of Medicine. Universidad Central de Venezuela using the standard protocol [37] followed by ELISA [38].

In both kits, the provider suggested that the data be calculated based on the optical density and that units define the value according to the formula. Using the controls established in our laboratory, positive samples were >12 units.

Patient (mean) absorbance value × 10 = [Units = U] Absorbance of the Cut-off

The analysis of IgG against *S. stercoralis* was performed using the Euroimmune ELISA kit following the automated version. The results were only defined by positive or negative results, confirmed by the proper controls from the kit and positive and negative samples from the Institute of Tropical Medicine, Faculty of Medicine, Caracas.

Total IgE was assessed using the ELISA kit from Thermo-Fisher, following the suggested protocol with minor modifications. The samples were calculated according to the instructions but then converted into IU/mL upon the request of the Ethical Committee, since the values were given to the patients and had to be in the same range as the other tests performed with other kits. The conversion was performed using the calculation from the web page at https://unitslab.com/.

### 2.3. Analysis of Stool Samples to Certify Infection of A. lumbricoides and S. stercoralis

The stool samples were analyzed using the Kato–Katz method as a standard, followed by the FLOTAC^®^ method as described before [35]. The Kato–Katz method was less sensitive for *Ascaris lumbricoides* than the FLOTAC^®^ method (85% matching), as described in the literature [36]. No difference was observed with *Strongyloides stercolaris*. Coinfection of *Ascaris lumbricoides* and *Strongyloides stercolaris* was observed using both methods.

### 2.4. Statistics

The results were analyzed using the Graph Prism Software version 5.0. One-way ANOVA was used to compare the different groups. The Chi-square test with Yate’s correction was used to define the frequency of infection. In addition, the Pearson coefficient was used to calculate the quantitative values. The statistical difference was set to *p* < 0.05.

## 3. Results

The general characteristics of the population are illustrated in Table 1. The control group is composed of nonsmoker individuals. The gender was similar in the four groups. The age difference was due to the difficulty of finding proper controls without comorbidities above 60 years.

Table 2 presents the hematological values for the different groups. Significant differences (*p* < 0.001) were observed in the percentages of neutrophils and eosinophils, while no variations were noted in the percentages of total lymphocytes or monocytes.

In Table 3, the frequency of parasite infection is depicted. It is observed that atopic individuals with elevated IgE levels exhibited the highest prevalence of *Al* infection, with four patients testing positive for *Tc.* Notably, significant disparities in the frequency of *Al* infection were noted compared with the control group (*p* < 0.0001). *Ss* infection was not observed in these groups.

In Table 4, the data present the prevalence of parasite infections among individuals with asthma, COPD, and atopy. Upon comparison, no significant variance in infection frequency was found between asthma and COPD. However, both groups exhibited significantly different infection frequencies than the atopic group (*p* < 0.0001). The occurrence of positive antibodies to TC was similar across the asthma, COPD, and atopy groups. Only four COPD patients tested positive for antibodies against *Ss*, showing a significant difference from the other three groups (*p* < 0.01). Additionally, four individuals from the atopic and asthma groups tested positive for *Al* and *Tc*. Based on confirmatory tests, it is highly likely that both parasites infected these individuals.

In Table 5, we examined the correlations among age, IgE, FEV1, eosinophil counts, and ELISA values to investigate potential associations with parasite infection. In the control group, despite normal parameters, a correlation was found between eosinophil values and the IgG titers for *Al*. No correlations were observed with the remaining parameters. As anticipated, IgE values were correlated with VEF1 and eosinophil values in the atopic group, yet no correlations were found with parasite IgG titers. In the asthma group, the most significant correlations were identified; age, IgE, and VEF1 were all found to be significant. Notably, IgG titers against *Al* were associated with IgE, eosinophil numbers, and VEF1, while IgE and eosinophil values were linked with IgG titers for *Tc.* In the COPD group, the only significant difference observed was between eosinophil and IgE values.

## 4. Discussion

Patients with atopy, asthma, and COPD present complex cases due to the predisposition to multiple medical conditions associated with chronic disease. The use of steroids as a standard anti-inflammatory treatment has led to various secondary effects, thereby limiting the effectiveness of treatment. Conversely, the approach to assessing, diagnosing, and treating various obstructive diseases has evolved in recent years.

In tropical countries, especially in underdeveloped areas, there are high frequencies of parasitic diseases, and it is difficult to find individuals who are not infected. The present report studied three parasites that migrate to the lungs at some point in their life cycle. It is well known that *Strongyloides stercolaris* infection can persist in the host for a long time [23] and that *Ascaris lumbricoides* may persist in immunosuppressed patients [17,41]. *Toxocara canis infection* is more common in toddlers than elders; however, contact with infected animals, usually puppies, exposure to contaminated soil, or consumption of undercooked or raw meat from intermediate animals is possible, especially in the countryside [28]. In all cases, lung migration upon or over-infection may be observed. This migration may increase susceptibility to inflammation and inflammation induced by allergens, cigarette smoke, or other gases. Several reports of increased susceptibility to long bronchoconstriction have been reported along with parasitic infection [17,20,23,28,42,43,44].

The study presented here was composed of different groups. The control group of atopic individuals was included to analyze the possible role of parasite infections in FEV1 and eosinophil counts. As expected, the IgE levels were high and can be considered an essential marker for analysis. Individuals with high IgE levels and atopic conditions showed no signs of lung obstruction, indicating that the Th2 pattern was only involved in the allergic reaction. IgE levels were correlated with FEV1 in the asthmatic and atopic groups, suggesting that IgE is unrelated to bronchoconstriction. Some studies have sought to establish a connection between IgE levels and the severity of asthma symptoms, proposing that parasites may be responsible for the severity of bronchoconstriction [44,45,46,47]. However, based on the results of our study, it is evident that the contribution of IgE is only partial, as demonstrated by the atopic group, where IgE levels were higher than in the asthmatic group. Probably, as other authors have suggested, IgE is a consequence of Th2 activation [47], and it may help differentiate different clusters of patients responding to antigens [48].

It is of interest to observe that only four individuals diagnosed with asthma exhibited positive IgG titers for *Al* and *Tc*. These patients reside in rural areas, where they are in close proximity to animals and have restricted access to clean water. Following treatment, their stool tested negative for *Al*, and their asthma symptoms showed improvement during the follow-up visit. Nevertheless, these results cannot be conclusive due to the low number of individuals.

Interestingly, only a correlation was observed in COPD patients, referring to IgE titters and eosinophil numbers. Recently, eosinophil values have been considered critical in COPD treatment [6,7,8]; even though there are no other correlations, the values were vital to analyzing patient treatment. Patients with parasitic infection were treated, but only minor changes were observed in VEF1. We cannot discard that some of the COPD patients had asthma-COPD overlap syndrome due to the increased amount of IgE and probably other allergic conditions that the treatment may mask.

There could be two reasons for the lack of correlations observed in the COPD group. First, age might make individuals less responsive to the allergen. Additionally, the anti-inflammatory treatment could reduce the response. Parasite infection may be confused with exacerbation in COPD. Toychiev A. et al. [49] conducted a study to examine the impact of *Ascaris lumbricoides* on the development of chronic pulmonary aspergillosis in patients with COPD. They discovered that the prevalence of *A. lumbricoides* in COPD patients with chronic pulmonary aspergillosis is high. Furthermore, the study found elevated levels of IL-1β and TNFα, indicating that ascariasis increases susceptibility to *Aspergillus* sp. in COPD patients.

Smoking may also affect IgE levels, as documented in [50], and eosinophil involvement in the airways [51]. How smoking affects the migration of the parasites and the possible allergic response in the lung tissue is unknown. Nonetheless, in asthmatic patients, steroids may facilitate parasite infection and progression [44], and biological therapy may also affect the immune response against pathogens [52]. The link between environmental factors and therapy in COPD requires further research.

The presence of parasite infections may play a significant role in various chronic inflammatory conditions, potentially contributing to an increase in symptoms but not necessarily essential to the progression of the disease. Investigating the long-term effects of antiparasitic treatment on these individuals would be valuable. Better control of parasitic diseases could improve patients’ quality of life.

Understanding the underlying rationale for parasitic infections is crucial, whether they stem from environmental or household exposure, a compromised immune response due to anti-inflammatory therapy in conjunction with the chronic nature of the disease, or are acting as an independent contributing factor. In tropical countries, parasite infection is common, especially in poor areas, due to a limited water supply. At least 30% of our cohort lives in poor areas of the city or the countryside; however, we could not find a correlation between low income and frequency of infection. Antibodies may be a valuable marker to define specific infections. Since infection by *Ascaris* and *Strongyloides* may be long-lived without clinical manifestations, it is unclear if pharmacological treatment is efficient in decreasing the parasite burden and is sufficient to prevent reinfection.

## 5. Conclusions

*Ascaris lumbricoides* infection is common in the Venezuelan population; however, it is more prevalent in atopic patients and moderate-to-severe patients with asthma or COPD. *S. stercolaris* infection is less frequent and only observed in this cohort of COPD patients. *T canis*-specific IgG was observed in around 10% of patients but not in controls. The higher incidence of parasite infection in patients may be due to partial-to-severe immune-compromised responses, which may be due to treatment or other medical conditions, especially in COPD patients. Routine screening for parasite infection in atopic, asthmatic, and COPD patients is highly recommended.

## Figures and Tables

**Table 1:** The general characteristics of the cohort are shown.

	CONTROL	ATOPIC	ASTHMA	COPD	*p*
n	100	100	100	100	ANOVA
AGE	41.13 ± 15.6	46.6 ± 16.9	50.2 ± 13.5	65.7 ± 7.0	>0.01
FEMALE (%)	57	54	58	56	0.9
SMOKERS (%)	0	10	10	75	>0.0001
FVEF_1_% pred	110.33 ± 6.8	102.9 ± 5.9	76.7 ± 12.3	58.8 ± 9.1	>0.0001
FEV1/FVC ratio	85.8 ± 4.5	80.6 ± 3.2	69.1 ± 11.9	53.6 ± 12.6	>0.0001
IgE (IU/mL)	68.2 ± 16.2	321.8 ± 150.8	199.4 ± 147.1	155.2 ± 129.3	>0.0001
% elevated IgE	0	89	61	39	>0.0001

The table represents the characteristics of the four different groups. One-way ANOVA was used to ascertain statistical differences between the groups.

**Table 2:** Hematological values of the different groups.

%	CONTROL	ATOPIC	ASTHMA	COPD	ANOVA
Lymphocytes	28.8 ± 2.5	30.0 ± 2.4	29.6 ± 3.0	29.2 ± 3.4	0.9
Neutrophils	63.6 ± 3.0	56.0 ± 4.4	54.4 ± 3.2	59.4 ± 2.1	>0.001
Monocytes	6.3 ± 1.6	7.1 ± 1.4	7.2 ± 1.8	6.9 ± 1.9	0.5
Eosinophils	1.3 ± 1.2	7.0 ± 1.5	5.7 ± 4.3	4.6 ± 3.0	>0.0001

The table represents the hematologic values of the four different groups. One paired ANOVA was performed for each type of leukocyte. The percentage of neutrophils was lower in the atopic and asthma groups than in the controls and COPD groups. The proportion of eosinophils was higher in the three patient groups compared with the controls.

**Table 3:** Incidence of confirmed parasitic infections in the control and atopic group with normal IgE levels.

	IgE	n	*Al*	*Ss*	*Tc*
CONTROLS	Normal	100	6 *	0	0
ATOPIC	Normal	11	1 *	0	0
	High	89	18 *	0	4 **

Table legend: * stool tests confirmed infection, ** IF and ELISA tests confirmed infection. *Al* states for *Ascaris lumbricoides*, *Ss Strongyloides stercolaris* and *Tc* for *Toxocara canis*. Only one atopic patient was positive for *Al* and *Tc*.

**Table 4:** Incidence of confirmed parasitic infections in the asthma and COPD groups.

ASTHMA		N	*Al*	*Ss*	*Tc*
	Severe	10	5	0	1
	Moderate	48	7 *	0	5 **
	Intermitent	28	0	0	0
	Mild	14	0	0	0
COPD	Severe	13	1	3	0
	Moderate	77	8 *	1 *	4 **
	Mild	10	0	0	0

The table represents the number of patients categorized, as described in the Material and Methods section, and the number of individuals with positive IgG. *Al* is *Ascaris lumbricoides*, *Ss* is *Strongyloides stercolaris*, and *Tc* is *Toxocara canis*. * Stool tests confirmed infection; ** IF and ELISA tests confirmed infection. Four asthmatic patients were positive for *Al* and *Tc*. None of the COPD patients were positive for more than one parasite. Patients with severe or moderate disease have a higher incidence of parasite infection, statistically significant for asthma (*p* < 0.01) but not for COPD.

**Table 5:** Correlations between the titer of IgG and FEV1 values.

Controls	FEV1	IgE	Eo	*Al* IgG Values	*Tc* IgG Values
Age	−0.31	−0.11		−0.01	−0.02
IgE	−0.02		−0.02	−0.08	−0.11
VEF1			−0.2	−0.07	−0.09
Eo				0.52	−0.14
Atopic					
Age	−0.64	0.32		0.36	0.18
IgE	−0.48		0.73	0.32	0.08
VEF1			−0.15	0.2	0.13
Eo				0.17	0.08
Asthma					
Age	−0.37	−0.06		−0.02	−0.03
IgE	−0.44		0.86	0.55	0.46
VEF1			−0.52	0.49	0.3
Eo				0.55	0.4
COPD					
Age	−0.27	0.2		0.17	0.18
IgE	−0.16		0.82	0.06	0.32
VEF1			−0.29	−0.2	−0.05
Eo				0.1	0.23

The table represents the Pearson correlations encountered with the different categories analyzed in the cohort.

## Data Availability

The raw data supporting the conclusions of this article will be made available by the authors on request.
